# Investigation of Electrochemical Assisted Deposition of Sol-Gel Silica Films for Long-Lasting Superhydrophobicity

**DOI:** 10.3390/ma16041417

**Published:** 2023-02-08

**Authors:** Baoming Zhou, Yongling Wu, Hongyu Zheng

**Affiliations:** Centre for Advanced Laser Manufacturing (CALM), School of Mechanical Engineering, Shandong University of Technology, Zibo 255000, China

**Keywords:** sol-gel, electrochemical assisted deposition, superhydrophobicity, self-cleaning, contact angle

## Abstract

Current methods for the protection of metal surfaces utilize harsh chemical processes, such as organic paint or electro-plating, which are not environment-friendly and require extensive waste treatments. In this study, a two-step approach consisting of electrochemical assisted deposition (EAD) of an aqueous silane solution and a dip coating of a low surface energy silane for obtaining a superhydrophobic self-cleaning surface for the enhanced protection of copper substrate is presented. A porous and hierarchical micro-nanostructured silica basecoat (sol-gel) was first formed by EAD of a methyltriethoxysilane (MTES) precursor solution on a copper substrate. Then, a superhydrophobic top-coat (E-MTES/PFOTS) was prepared with 1H,1H,2H,2H-Perfluorooctyltriethoxysilane (PFOTS) for low surface energy. The superhydrophobic coating exhibited anti-stain properties against milk, cola, and oil, with contact angles of 151°, 151.5°, and 129°, respectively. The EAD deposition potential and duration were effective in controlling the microscopic morphology, surface roughness, and coating thickness. The E-MTES/PFOTS coatings exhibited chemical stability against acids, bases, and abrasion resistance by sandpaper. The proposed 2-layer coating system exhibited strong chemical bonding at the two interfaces and provided a brush-like surface morphology with long-lasting superhydrophobicity. The developed method would provide an environment-friendly and expedient process for uniform protective coatings on complex surfaces.

## 1. Introduction

Metal surfaces are prone to oxidation and corrosion in ambient conditions and require surface protection. Currently, surface coatings such as organic paint and metallic coatings (e.g., nickel and chrome) by electroplating processes [1] are applied to provide protection. However, the electroplating process uses harsh chemicals such as strong acids and bases, which are environmentally unfriendly. The process creates some hazardous waste products, which can severely damage the environment if they are not properly disposed of [2]. Therefore, alternative environmentally friendly processes with enhanced surface protection functionality are in high demand with urgency. Sol-gel coatings are made from hydrolyzed solutions of organometallic precursors, typically silica-based materials, which are chemically stable without harmful substances, and provide environmentally friendly surface protection. However, under harsh environments, sol-gel thin-film coating alone may be insufficient for surface protection. Inspired by the self-cleaning effect of lotus leaves [3], self-cleaning coatings are expected to repel liquids (including water, acids, bases, and solvents) from contacting the metal surfaces and, therefore, further enhance surface protection function. Superhydrophobic surfaces with water contact angles >150° have been shown to exhibit the self-cleaning effect and are studied for a wide range of applications [4]. Various strategies for constructing superhydrophobic surfaces on metal substrates have been investigated in the past decades [5,6]. Techniques for surface modification include chemical etching [7], laser patterning methods [8,9], wet etching [10], Chemical Vapor Deposition (CVD) [11], micro-arc oxidation (MAO) [12], and surface coating of intrinsically low energy materials, such as PDMS, fluoropolymers, and fluorinated silanes [13,14,15,16]. These methods either employ expensive machines or use harsh chemicals to change the metal surface conditions. These complex or environmentally unfriendly processes have hindered further widespread applications of protective self-cleaning coatings for metals, especially for 3D complex surfaces. Superhydrophobic coatings are usually prepared by a two-step process, where a surface with sufficient roughness is firstly constructed by various physical and chemical methods [17,18,19,20] and then further modified with low surface energy materials. Sol-gel methods [21,22] are increasingly adopted for the preparation of superhydrophobic surfaces due to the wide range selection of precursors, the relative simplicity of the wet chemical process, and the use of inexpensive and environmentally friendly raw materials. Conventionally, silica-based sol-gel coatings are applied by dip-coating, spray-coating, roller-coating, or spin-coating [23,24] processes. The driving force for the generation of thin films by these self-assembly-like methods relies heavily on the degree of the hydrolytic condensation reaction of the sol-gel monomer [25]. For this reason, it is difficult to deposit thicker silica films uniformly, especially on 3D complex industrial parts [26,27]. The inherent mechanical properties and weak adhesive strength of inorganic sol-gel coatings on metal substrates have restricted their industrial applications as a pre-treatment layer for coating systems.

EAD is a recently developed method for the preparation of coatings on conductive materials based on the sol-gel principle [28] and allows for the preparation of thicker and rougher sol-gel films. EAD is based on the principle of in-situ catalysis of sol-gel chemistry (hydrolysis and condensation reactions) by the application of negative potential. The immediate condensation of the solute on the electrode surface can be accelerated by the reduction of oxygen or some specific ions supporting the electrolyte or by hydrolysis, which leads to the increase of pH value near the electrode surface. The chemical reaction formula in this process is expressed as Equations (1)–(3) [29,30].

2H_2_O + 2e^−^ = 2OH^−^ + H_2_ (1)

O_2_ + 2H_2_O + 4e^−^ = 4OH^−^ (2)

NO_3_ + H_2_O + 2e^−^ = NO^2−^ + 2OH^−^ (3)

**Figure 1:**
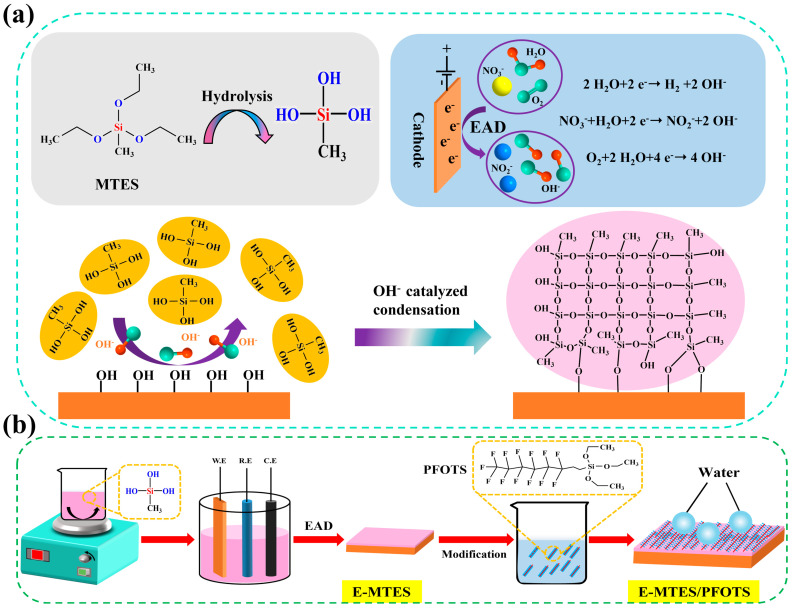
Schematic diagram of (**a**) hydrolysis reaction of MTES and EAD of catalytic condensation-reacted silica films on Cu substrates and (**b**) two-step EAD for the preparation of superhydrophobic E-MTES/PFOTS films.

A schematic diagram of the described reaction process is shown in Figure 1a. It is worth noting that during the above “electrodeposition” process, the silane component does not lose or gain electrons at the electrode surface and the pH value of the bulk solution itself does not change throughout the process, and no new substances are introduced [31,32,33]. Compared to conventional self-assembled films, these electrodeposited films (e-films) have several unique advantages: (i) The base catalysis near the cathode surface provides an additional driving force for film formation, resulting in thicker and rougher films [34,35]; (ii) Gelation is separated from solvent evaporation and occurs during electrodeposition, producing films with greater porosity and better intra-film cohesion; (iii) The derived OH- ions also catalyze the chemical bonding process between the sol-gel film and the substrate [36]. EAD has the significant advantage of depositing coatings on complex non-planar geometries and controlling the thickness and composition of the nanocomposite and ensuring the surface structures are ohmically connected to the metal substrate. Mandler et al. [37,38] demonstrated the electrochemical co-deposition of sol-gel monomer with copper ions or gold nanoparticles leading to the formation of thin nanocomposite films. Their results showed that the ratio of sol-gel monomer to copper ions/gold nanoparticles strongly influenced the morphology and grain size of the films. This method demonstrated the general applicability of EAD in templating nano-sized objects in thin films. Electrodeposited silica-based films are often used in research on porous electrode materials, electrochemical sensors, biodegradable polymer films, and the preparation of superhydrophobic films [39,40,41].

Compared with conventional electrochemical plating processes, EAD requires lower energy input and no need for waste treatment. Therefore, EAD offers a low-cost and environmentally friendly process. It is suited for producing relatively thicker and rougher films uniformly on complex conductive surfaces and thus provides a new and convenient method for preparing superhydrophobic surfaces. The construction of superhydrophobic surfaces can be realized by the one-step method or two-step method. The former is achieved by one-step deposition of inorganic and organic composite film from a mixed silane precursor solution with various fillers [42]. The high content of organosilane components in the one-step superhydrophobic films tends to cause weaker mechanical properties than pure inorganic silica films. The latter is obtained by the EAD of silica films followed by low surface energy modification with long alkyl chain organosilanes or fluorosilanes [43]. Wang et al. [44] prepared superhydrophobic membranes with a water contact angle of 153.5 ± 3° in a two-electrode cell using a synthetic 3-undecyl-4-amino-5-mercapto-1,2,4-triazole (UAMT) in a one-step electrolytic method using copper foil as the anode and platinum wire as the cathode. Wu et al. [45] reported the deposition of silica films at a typical constant potential of −1.3 V on 306 L stainless steel (SS) or indium tin oxide (ITO) glass electrodes, followed by hydrophobic modification with dodecyltrimethoxysilane (DTMS) to obtain excellent water repellency. Further, Hu et al. [46] mixed dodecyltriethoxysilane (DTMS) and tetraethoxysilane (TEOS) sol-gel precursors and prepared SiO_2_/DTMS hybrid sol-gel superhydrophobic films on mild steel substrates by a one-step electrodeposition method. A two-step method to create superhydrophobic surfaces on mild steel was reported by Zhang et al. [43]. First, highly porous and graded nano/microstructured silica films were prepared by EAD, and then were further treated with long alkyl chain dodecyltrimethoxysilanes. The superhydrophobicity provided by the rigid SiO_2_ matrix showed high mechanical durability against abrasion and good repair ability for thermal damage treatments. Despite the above-mentioned methods, the combination of EAD and self-assembly of the sol-gel top layer to obtain superhydrophobicity on the copper substrate has not been systematically investigated.

In this work, a two-step method is studied for the preparation of superhydrophobic coatings on a copper substrate. The first step is to generate a rough micro/nanostructures sol-gel coating on Cu substrates from MTES precursor solutions by EAD. The effects of key EAD parameters including deposition potential and duration on the surface morphology and thickness of the films are investigated. The second step is to modify the sol-gel coatings by fluorination with PFOTS to obtain superhydrophobic surfaces with low surface energy. The coating stability, durability, and self-cleaning ability are compared to those prepared by the one-step method. It is aimed to provide an environment-friendly and expedient process for uniform protective coatings.

## 2. Experimental

### 2.1. Materials and Chemicals

A commercial copper sheet with a thickness of 0.3 mm (supplied by Gaosheng Instrument Ltd., Dongguan, China) was cut into 15 mm × 50 mm-sized substrates. After sanding with 1000# sandpaper, cleaning with anhydrous ethanol ultrasonically for 5 min, and blowing dry with nitrogen gas, it was ready for experiments. The copper material composition is shown in Table 1.

Methyltriethoxysilane (MTES) (98%, Shanghai Mackin Biochemical Co., Ltd., Shanghai, China) and potassium nitrate (KNO_3_) (99%, Sinopharm Chemical Reagent Co., Ltd., Shanghai, China) were used without further purification. The precursor solution (pH = 5–6, adjusted by CH_3_COOH) for electrodeposition consists of 100 mL 0.2 M KNO_3_, 200 mL ethanol, and 50 mL MTES. Firstly, MTES and ethanol solution were mixed and stirred, and then an aqueous solution of KNO_3_ was slowly added to the mixed solution in the previous step, and finally, the solution was hydrolyzed for 6 h at 25 °C under vigorous stirring. Before electrodeposition, for easy characterization of samples, the copper plates were taped at one side surface with an insulating tape leaving the other side for coating deposition. Figure 1b shows a schematic diagram of the preparation process of E-MTES films and fluorination-modified superhydrophobic surfaces.

### 2.2. Electrodeposition of E-MTES Films

The electrodeposition was carried out on a Vertex. 5A electrochemical working station (IVIUM instrument, Eindhoven, The Netherlands), using a conventional three-electrode compartment. A Graphite Rod (diameter 5 mm × length 120 mm) was used as the counter electrode and a commercial Ag/AgCl electrode (in saturated KCl solution) as the reference. Silica sol-gel film was deposited onto the copper substrate from the precursor solution by applying a cathodic potential (−1.0 V, −1.1 V, −1.2 V, and −1.3 V),for different time duration (100 s, 200 s, 400 s, 600 s, 1000 s). After deposition, the copper sheet was slowly lifted at a speed of 8 mm/s and put into the oven for drying at temperature of 80 °C for 10 min. For comparison, dip-coated silica films were also prepared by immersing the substrate directly into the silane solution for different times (100 s, 200 s, 400 s, 600 s). For all conditions, three replicate samples were prepared.

### 2.3. Preparation of Superhydrophobic Composite Coating

To obtain a superhydrophobic surface, surface fluorination treatment was conducted. The solution for the hydrophobic modification was composed of 2.5 g 1H,1H,2H,2H-Perfluorooctyltriethoxysilane (PFOTS), 35 g propanol, and 0.2 g of hydrophilic nano-silica particles. The resulting solution was pre-hydrolyzed at 35 °C for 24 h to form a fluorosilane sol-gel solution. The sample with electro-deposited coating (E-MTES) was then dipped into the fluorosilane sol-gel solution by a simple dip-withdraw coating process at a constant rate of 6 mm/s. After immersed in the sol-gel solution for about 30 s, the sample was withdrawn at the same constant rate, and then cured in an oven at 100 °C for 1 h. The finally obtained superhydrophobic coating is named E-MTES/PFOTS.

### 2.4. Characterization

The morphology of the coated surface was characterized by field emission scanning electron microscopy (FE-SEM) (QUANTA 250 FEG, ThermoFisher Scientific, Waltham, MA, USA) at an accelerating voltage of 20.0 kV. To obtain high-resolution FE-SEM images, the surfaces were sputtered with a gold layer using a thermal evaporator to improve electrical conductivity. The chemical composition of the sample surface was analyzed by an energy-dispersive X-ray spectrometer (EDS). The chemical structure of the coating surface was analyzed by Fourier transform infrared spectroscopy (FTIR) (Nicolet 5700, Thermo-Nicolet Instruments, Waltham, MA, USA). The FTIR spectrum was obtained in the infrared region from 700 to 4000 cm^−1^. All the spectra were recorded at 2 cm^−1^ resolution. The scans were repeated twice to ensure repeatability. The coating surface chemical composition was determined by monochromatic Al Kα (1486.6 eV) x-ray photoelectron spectroscopy (XPS, Thermo Kalpha). The static water contact angle was measured by a contact angle instrument (JC2000D1, Zhongchen, Shanghai, China). The water droplet size used for the measurements was 4 μL. The contact angles were obtained by averaging the measured values at three different locations on the inspected surface. The three-dimensional morphology and surface roughness of the samples were obtained using an optical profilometer (UP-WLI, RTEC instrument, San Jose, CA, USA). The average roughness was measured on an area of 0.6 × 0.4 mm^2^ in non-contact mode at 20 X magnification. For each sample, readings were taken in at least three different positions and the average value was taken as the final value. The bonding strength between the coating and substrate was measured by the cross-hatch testing according to ASTM D3359 standard method [47].

## 3. Results and Discussion

### 3.1. Process Control of the EAD of E-MTES Coatings

**Figure 2:**
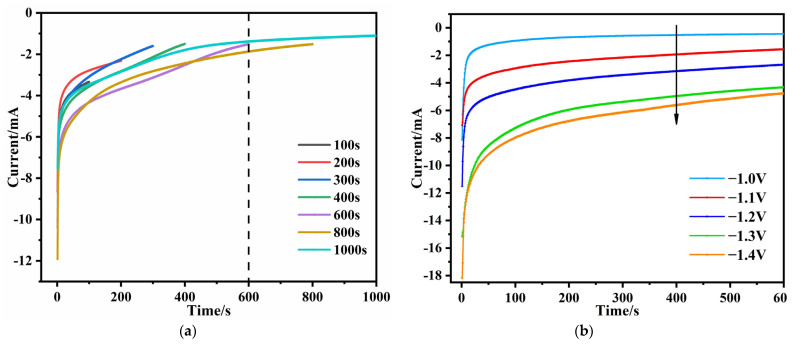
(**a**) Current-time response curves for the process of depositing silica films on Cu at different times at −1.2 V vs. Ag/AgCl. (**b**) Current-time response curves for the deposition of silica films at different voltages (scan rate: 1 mV/s).

During the electro-deposition process, the time and current were recorded for each coating sample. Figure 2a shows the correlation between the deposition current and the different deposition time under a typical deposition voltage of −1.2 V vs. Ag/AgCl. Due to the double layer charging process at the electrode and solution interface, the potential-step curve [48] was used in this setup. The current increased sharply during the first few seconds of recording and then gradually reached a quasi-steady state value. It was also noticed that the current response curve flattened when the deposition time reached 600 s. This indicated the completion of the film growth on the substrate. The current-time relationship of the samples at different voltages was studied using 600 s as a threshold value. As shown in Figure 2b, with the potential absolute value increased from −1.0 V to −1.4 V, the current curve shifted to more negative values. The higher current densities and the higher applied potentials led to a larger amount of hydroxide produced and, thus, the higher pH values at the electrode surface and increased the rate of film formation. Therefore, the film thickness can be adjusted by varying the potential of the working electrode, which controls the film formation rate.

### 3.2. Surface Microstructure and Coating Thickness by EAD

**Figure 3:**
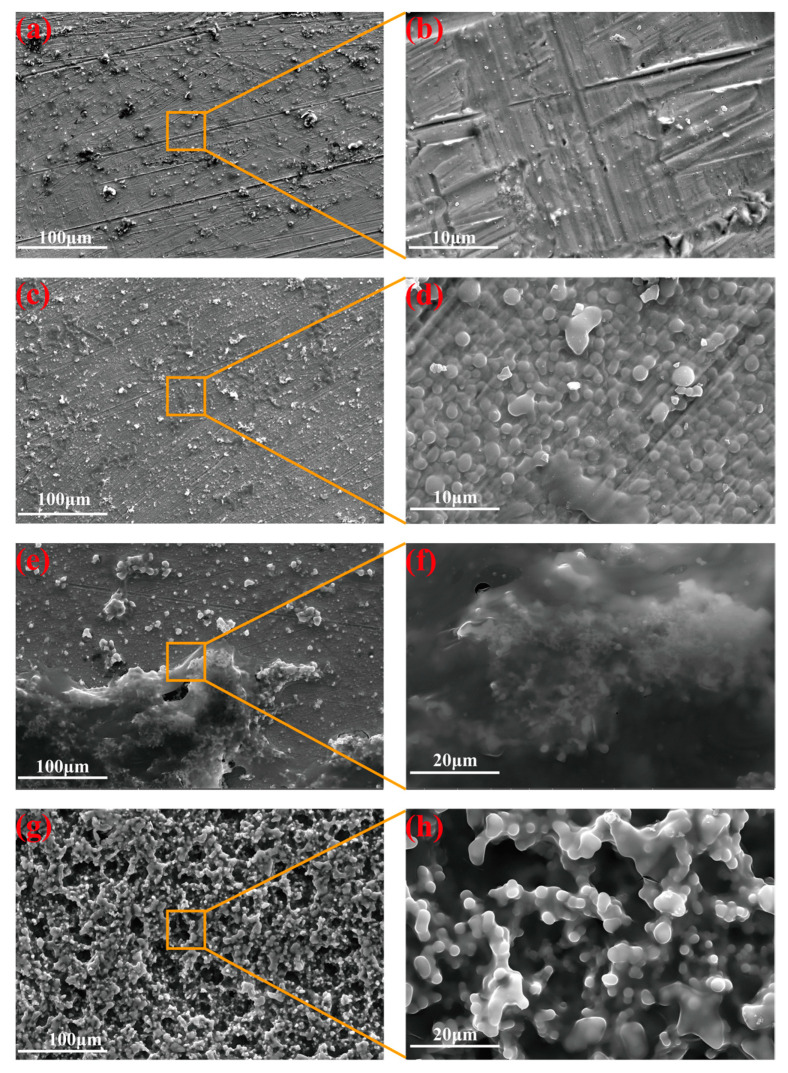
FE-SEM images of the top-view of the silica films deposited on Cu with different deposition times (applied potential at −1.2 V vs. Ag/AgCl): (**a**,**b**) 100 s; (**c**,**d**) 200 s; (**e**,**f**) 400 s; (**g**,**h**) 600 s.

The deposition process of sol-gel silica films depends on various parameters, such as deposition time and applied potential. Figure 3 shows the FE-SEM images of the top-view of the E-MTES coating on Cu substrates, showing that film deposition took place consecutively when prolonging the duration at the applied potential of −1.2 V vs. Ag/AgCl. The FE-SEM results showed that the surface morphology of the film changed with increasing deposition time. At a shorter deposition duration (Figure 3a,b), the surface was relatively flat, and there were still visible polishing marks on the copper surface. This was due to the incomplete growth of the film, which had not yet covered the polished marks on the Cu substrate. As the deposition time increased, the surface became rougher. At a deposition time of 200 s, the film consisted of a tightly packed arrangement of micro- and nano-sized silica spheres (Figure 3c,d). As the deposition time was increased to 400 s, the E-MTES films continued to grow, with flocculent polymers gradually covering the surface (Figure 3e,f), resulting in thicker films. This was attributed to more OH^−^ aggregation in the vicinity of the cathode, which facilitated the condensation process. When the time was increased to 600 s, the E-MTES films showed a layered morphology consisting of micro-structures and the films appeared highly porous (Figure 3g,h), leading to a further increase in the surface roughness. The film-growing principle of the EAD process consists of a few stages, from nucleation, growing into an island, further growing into continuous film, and then becoming porous due to the dissolution of the already formed film under prolonged immersion in the chemical solution (600 s). The rough and porous morphology facilitated the subsequent fluoro-silane surface modification to construct superhydrophobic surfaces. Cross-sectional SEM images and measurements of the coating thickness values at different deposition times: (a) 100 s, (b) 200 s, (c) 400 s, and (d) 600 s are shown in Figure S1. Table S1 lists the coating thickness values with varying deposition durations. The thickness of the coating increased exponentially with time. Similarly, the cross-sectional SEM images and measurements of coating thickness values at different deposition potentials: (a) −1.0 V, (b) −1.1 V, (c) −1.2 V, and (d) −1.3 V are shown in Figure S2.

**Figure 4:**
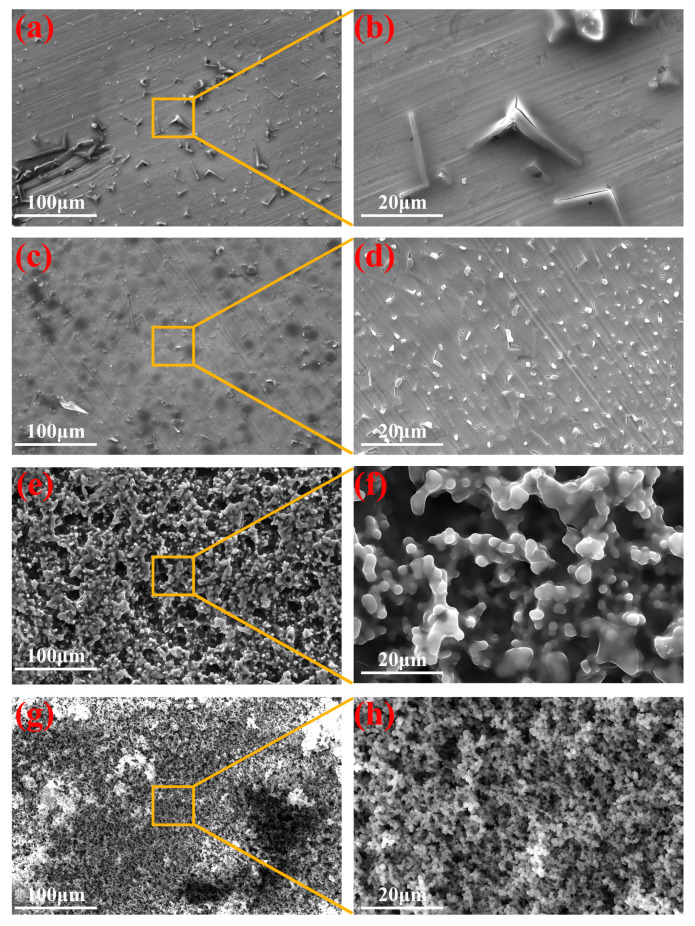
Top-view FE-SEM images of silica films prepared with different deposition potentials: (**a**,**b**) −1.0 V; (**c**,**d**) −1.1 V; (**e**,**f**) −1.2 V; (**g**,**h**) −1.3 V. (The deposition time was 600 s for all the samples).

**Figure 5:**
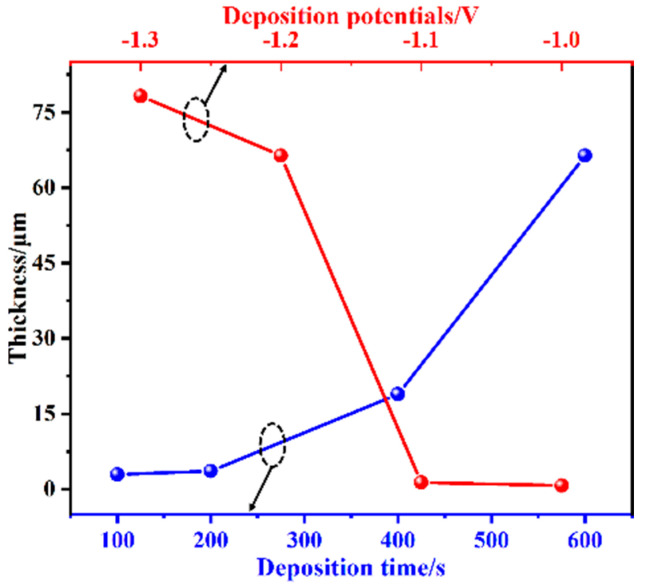
Influences of deposition time (blue) and potential (red) on the thickness of E-MTES films.

The effect of deposition potential on the coating surface morphology was studied using 600 s as a time reference for all the samples (Figure 4). Typically, organosilane precursors underwent hydrolytic condensation to produce silica nuclei and growing “islands” that gradually covered the substrate and formed the coating after curing. In this study, an initial “triangular” micro-structure was observed by EAD (Figure 4a,b) at −1.0 V, and the EDS elemental analysis (Figure S3) showed that the structure was mainly composed of Si and O elements. With increased deposition potential values, the micro-structure became finer (Figure 4c,d) and more inter-connected, as shown in Figure 4e–h. As the deposition potential became more negative, more OH^−^ ions accumulated at the cathode and at the same time accelerated the condensation process, resulting in a rougher and more porous E-MTES coating (Figure 4f,h). The coating thickness values were measured on the cross-sectional SEM images of the samples as shown in Figures S1 and S2. The thickness values were plotted against electrochemical deposition potential and deposition time as shown in Figure 5 and summarized in Table S2. It is seen from the curves and the values that with more negative deposition potential, the film thickness increased exponentially. Similarly, the film thickness increased with increasing deposition time.

### 3.3. Chemical Analyses of the Coated Surfaces

**Figure 6:**
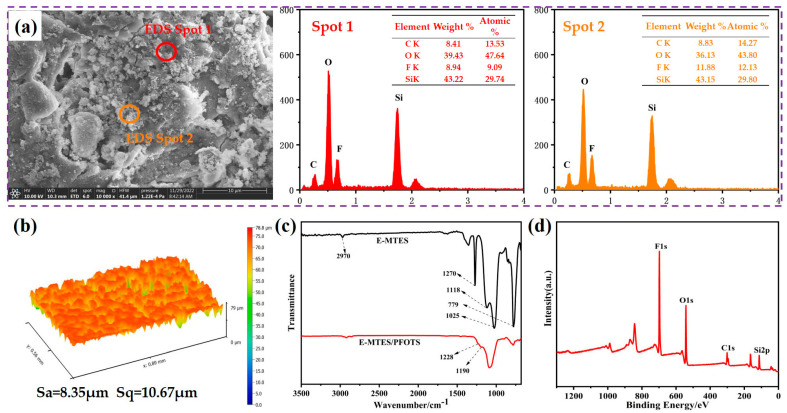
(**a**) FE-SEM image and EDS spectra of the superhydrophobic coating. (**b**) 3D morphology and roughness of superhydrophobic coating. (**c**) FT-IR spectrum of the E-MTES and E-MTES/PFOTS coatings, and (**d**) XPS spectrum of PFOTS coating surface (E-MTES parameters are −1.2 V vs. Ag/AgCl for 600 s).

**Figure 7:**
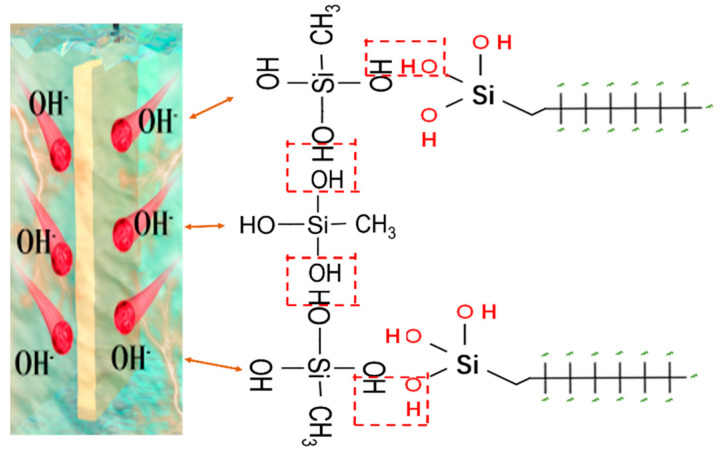
Illustration of the chemical bonds between the E-MTES layer and substrate and between PFOTS and E-MTES layers.

The coating surface with a deposition potential of −1.2 V vs. Ag/AgCl for 600 s was further analyzed. The FE-SEM image and EDS spectra and elemental mapping are presented in Figures S4 and S5. The E-MTES film contained a high amount of C, O, and Si, indicating that the coating had been successfully deposited onto the copper surface. The atomic weight ratios of C, O, and Si elements at different deposition times are shown in Figure 4a–d. The results indicate that the Si content of the coating increased with increasing deposition time, which was consistent with the results observed in FE-SEM (Figure 3). Figure 6 shows the surface profile analyses and chemical analyses of the superhydrophobic coating. It can be observed from Figure 6a that the surface became relatively smoother after fluorination modification, and the pores were “sealed.” Figure 6b shows the 3D morphology of the superhydrophobic coating, corresponding to Sa = 8.35 μm and Sq = 10.67 μm. The FT-IR spectra of the E-MTES and E-MTES/PFOTS coatings are shown in Figure 6c. The band at 2970 cm^−1^ is due to C–H stretching in CH_3_. The band at 1270 cm^−1^ corresponds to CH_3_ symmetric bending in Si-CH_3_ [40]. The main absorption peak at 1025 cm^−1^ corresponds to the asymmetric stretching vibration of Si-O-Si, which is the result of the silanol condensation reaction [49]. The results confirmed the formation of silica film on the copper substrate. With the increase of negative potential, the cathodic OH^−^ concentration increased, which greatly promoted the formation of well-grown cross-linked network chains with Si-O-Si bonds. The band at 1118 cm^−1^ is ascribed to Si-O-C. The band at 779 cm^−1^ shows that the condensation of Si-OH groups was almost completed and only a few Si-O-CH_2_CH_3_ groups remained [50]. The weak bands at 1228 and 1190 cm^−1^ observed on the superhydrophobic surface E-MTES/PFOTS coating were assigned to C-F stretching vibration. These results confirmed that PFOTS molecules had been successfully assembled on the porous E-MTES surface by the dip-coating method. To further confirm the formation of the PFOTS film, a high-resolution XPS spectrum of the PFOTS hydrophobic coating is shown in Figure 6d. The binding energy at 528.28 eV and 99.81 eV are O1s and Si2p, respectively. The strong energy peak at 683.88 eV represents elemental fluorine, which confirms the successful grafting of the PFOTS molecule. The fluorine modification is an important factor to achieve the desired superhydrophobicity. The elemental F energy peak was also observed in the two EDS spectra shown in Figure 6a, which were measured at two points on the superhydrophobic surface. The F atomic contents of these two points were about 10%, which further confirmed the result obtained by XPS.Figure 7 illustrates the chemical structure of each coating layer and the condensation reactions between hydroxyl groups of hydrolyzed silanes, which led to the formation of Si-O-Si bonds upon drying and condensation. This confirms the chemical bonding between layers and the brush-like fluoro-chains, which provided intrinsically low surface energy status. The coatings were subjected to a crosshatch adhesion test and achieved the grade “5B” (“5B” is the best grade of the adhesion test) according to ASTM D3359 [47].

### 3.4. Surface 3D Profile and Roughness

**Figure 8:**
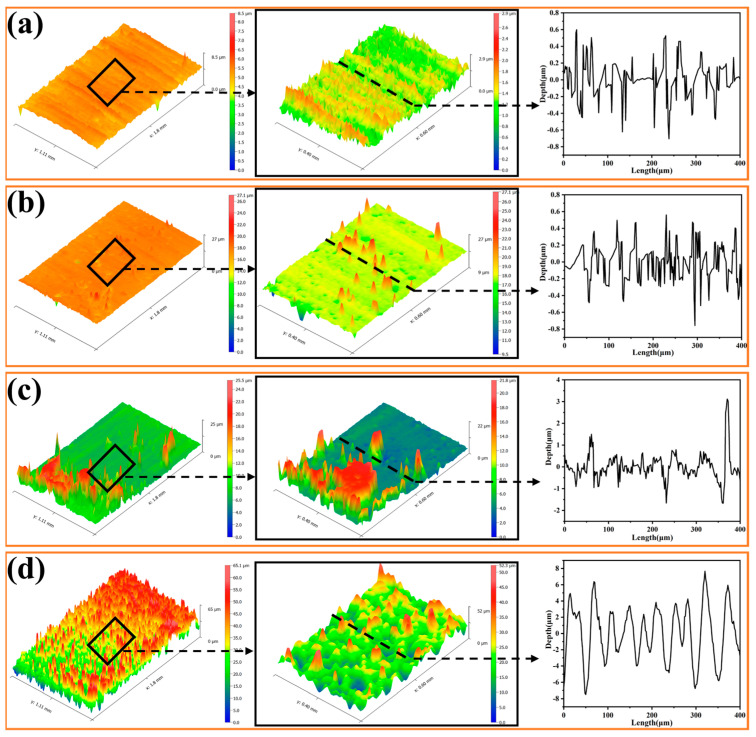
3D morphology and 2D profiles of E-MTES coatings at different deposition times: (**a**) 100 s; (**b**) 200 s; (**c**) 400 s; (**d**) 600 s. Roughness Sa values are 0.203 ± 0.0288 μm, 0.438 ± 0.079 μm, 2.17 ± 0.271 μm and 8.29 ± 0.829 μm, respectively.

**Figure 9:**
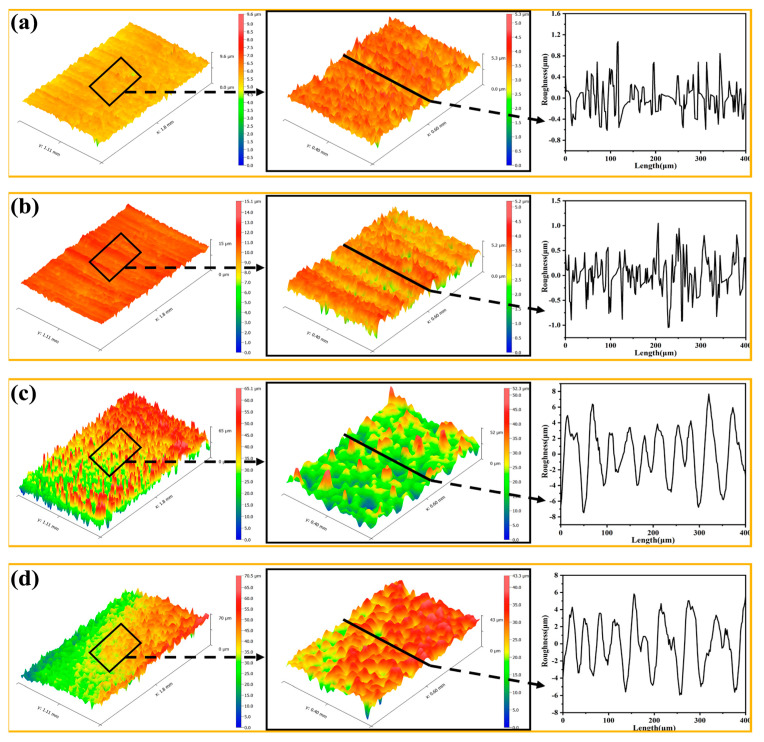
3D morphology and 2D profile of E-MTES coatings at different deposition potentials: (**a**) −1.0 V; (**b**) −1.1 V; (**c**) −1.2 V; (**d**) −1.3 V. Roughness Sa values are 0.306 ± 0.044 μm, 0.431 ± 0.017 μm, 5.01 ± 0.334 μm to 8.51 ± 0.244 μm, respectively.

**Figure 10:**
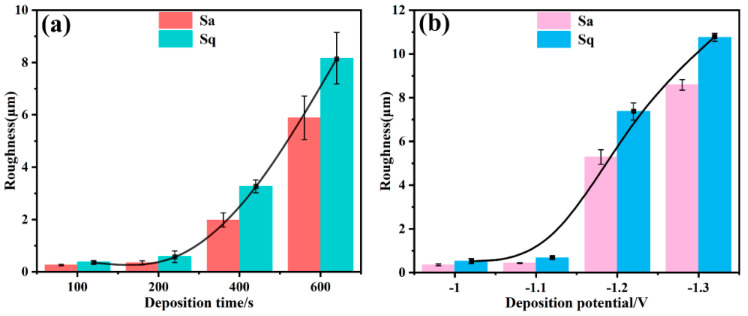
Surface roughness Sa and Sq values of the coatings versus (**a**) deposition time and (**b**) deposition potential.

It is well known that micro/nanostructures play a crucial role in the wettability of the coating surface. The surface roughness of the coating was assessed according to the deposition time and deposition potential. Figure 8 shows the 3D morphologies and 2D profiles of the sample surfaces after different deposition times. It is seen that when deposition time is short, the surface morphology is not uniform with the deposition occurring at some preferred area or points on the surface. With increasing deposition time to 600 s, the layer grew and gradually covered the whole surface, leading to a more regular and uniform surface structure. The increase in surface roughness was in good agreement with the FE-SEM images shown in Figure 3g,h, where the porous film was formed. Figure 9 shows the 3D morphologies and 2D profiles of the sample surfaces at different deposition potentials. It is seen that deposition potential was more effective in controlling the surface morphology. With deposition potential becoming more negative at −1.2 V and −1.3 V, the surfaces became rougher with more regular structures as shown in the 2D profiles of the surfaces in Figure 8d and Figure 9c,d, in which the peaks and valleys in the 2D profiles were more evenly distributed. Correspondingly, Figure 10 shows the average roughness (Sa) and the root means square roughness (Sq) of the surfaces. It is clear that as the deposition time increased and the deposition potential was more negative, the surface gradually became rougher, and the surface roughness increased almost exponentially. As shown in Figure 10, after increasing the deposition time from 100 s, 200 s, 400 s, to 600 s, Sa increased from 0.203 ± 0.0288 μm, 0.438 ± 0.079 μm, 2.17 ± 0.271 μm to 8.29 ± 0.829 μm, respectively, and Sq increased from 0.308 ± 0.056 μm, 0.833 ± 0.221 μm, 3.50 ± 0.246 μm to 10.56 ± 0.985 μm, respectively. Similarly, when depositing potential changed from −1.0 V, −1.1 V, and −1.2 V to −1.3 V, Sa increased from 0.306 ± 0.044 μm, 0.431 ± 0.017 μm, 5.01 ± 0.334 μm to 8.51 ± 0.244 μm, and Sq increased from 0.519 ± 0.114 μm, 0.636 ± 0.077 μm, 7.55 ± 0.392 μm, to 10.75 ± 0.185 μm, respectively. These results indicate that the EAD process parameters (e.g., time and potential) played a significant role in improving the coating surface morphology and facilitating the further formation of superhydrophobic surfaces. The regular distributed peaks and valleys on surface morphology acted as the anchoring force between the top-coat and base-coat and thus ensured strong adhesion between the two layers. By combining the strong chemical bonding between the silica basecoat and the fluorinated silane top-coat with the physical interlocking force, the 2-layer coating system is expected to provide long-lasting superhydrophobicity as well as mechanical durability.

### 3.5. Water Contact Angle (WCA) Measurements and Stability Tests

**Figure 11:**
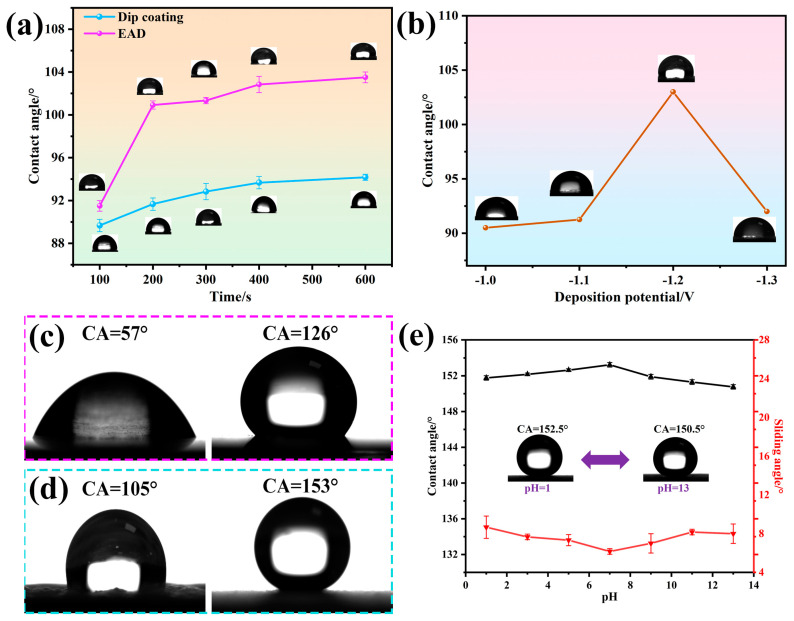
(**a**) Contact angles (CA) of coatings prepared by the EAD method (at −1.2 V vs. Ag/AgCl) and dip coating method at different times before fluorination modification. (**b**) The CA of coatings prepared at different deposition potentials before fluorination modification. WCA on Cu substrate: (**c**) Bare copper before and after fluorination modification; (**d**) E-MTES coating (prepared at −1.2 V vs. Ag/AgCl) before and after fluorination modification. (**e**) CA and sliding angle of E-MTES/PFOTS surface with water droplets of different pH values.

Contact angle measurement is the main method to evaluate the surface wettability of a solid material. The contact angles of the electrochemically deposited silicon films were measured by the Sessile drop method using a contact angle instrument [51]. Figure 11a shows the WCAs of the coatings made by EAD at different deposition parameters, with a comparison to the dip-coated surfaces at the same immersion times. It is seen that both coating methods showed increasing trends of WCAs with increasing deposition time, while EAD-coated surfaces exhibited higher WCAs (>100° after 200 s). Figure 11b shows the WCAs with varying EAD deposition potential. It shows a maximum WCA of 104° at −1.2 V. Figure 11c shows WCAs of bare copper before and after fluorination modification. Compared to the bare copper with a WCA of 57°, the fluorination-modified surface showed hydrophobicity (WCA of 126°). Figure 11d compares the WCAs of E-MTES coating (prepared at −1.2 V vs. Ag/AgCl) before and after fluorination modification. It shows that the EAD-coated surface had a WCA of 105°, while after fluorination modification, the WCA increased to 153°. This is attributed to the fact that the silane precursor MTES has a terminal group of -CH_3_, which is unable to produce long-chain (C-H) bonds. It is also difficult to obtain a superhydrophobic surface by relying only on the surface morphology of the E-MTES coating. The fluorination modification provided a long –CF chain at the outer surface of the EAD-coated rough surface, which enabled superhydrophobicity. The chemical stability of the superhydrophobic surface was evaluated by measuring the WCAs with water of different pH values ranging from 1 to 13, and pH adjustment was done using HCl and NaOH. As shown in Figure 11e, the WCAs of the E-MTES/PFOTS coating remained above 150° with a sliding angle of less than 10° at all times and the pH variation did not affect the WCA. The results demonstrated that the E-MTES/PFOTS coating had great resistance to both acidic and basic solutions.

The superhydrophobicity of the E-MTES/PFOTS coating confirmed the mechanism for producing superhydrophobic surfaces by combining surface morphology with low surface energy coating materials. When a liquid comes into contact with a rough superhydrophobic surface, it cannot fully penetrate into the surface structure but forms a heterogeneous wetting state with a composite interface combining solid, liquid, and air. The Cassie-Baxter model can be used to analyze the heterogeneous wetting state, and the equation is described as Equation (4) [52]:

(4) $$\cos\theta =\gamma_{S}f_{S}\cos\theta_{1}+(1-f_{S})\cos\theta_{2}$$

where $f_{S}\;$ describes the area fraction of the liquid−air interface occluded by the surface texture, and $\gamma_{S}$ describes the non-dimensional surface roughness factor of the superhydrophobic surface. θ is the real contact angle of the superhydrophobic surface, $\theta_{1}$ is the equilibrium contact angle on solid, and $\theta_{2}$ is the equilibrium contact angle on air. Since the value of the equilibrium contact angle on air is 180°, thus Equation (4) could be written as Equation (5) [53]:

(5) $$\cos\theta =\gamma_{S}f_{S}\cos\theta_{1}+f_{S}-1$$

Therefore, increasing the portion of the liquid-air interface and reducing the portion of the solid-liquid interface on a superhydrophobic surface can enhance its water repellency. The E-MTES coating’s highly porous micro-/nano-structure traps more air into the surface structure, thus increasing the fraction of the liquid-air interface. Therefore, after modifying PFOTS, the sample could reach a higher WCA.

### 3.6. Self-Cleaning Test and Durability Evaluation

**Figure 12:**
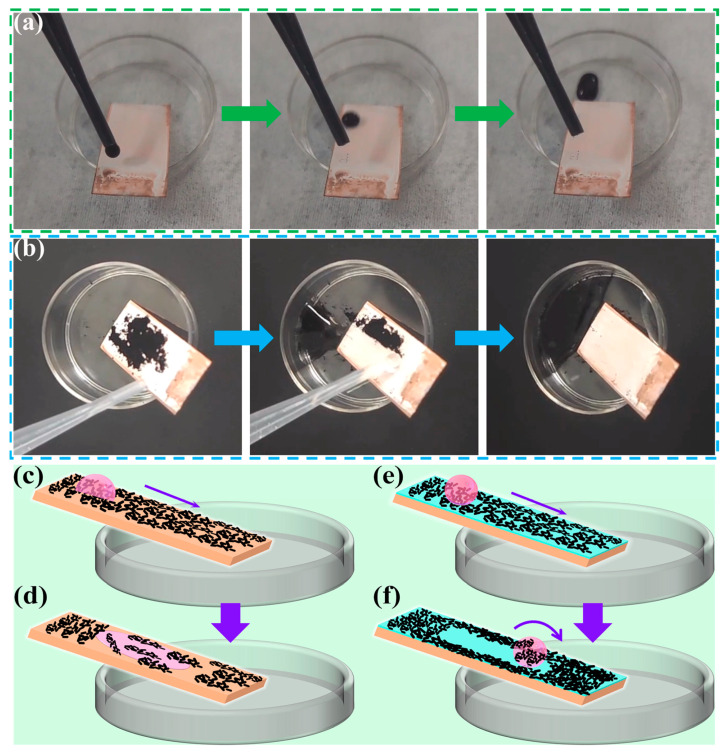
Self-cleaning tests of E-MTES/PFOTS surfaces. (**a**) The ink removal test on E-MTES/PFOTS surface. Self-cleaning testing process of the superhydrophobic surface by (**b**) carbon black powder. Self-cleaning mechanism of droplets on (**c**,**d**) bare copper and (**e**,**f**) superhydrophobic surface.

To demonstrate the self-cleaning property of the superhydrophobic surfaces, black ink was used to stain the surface. The contact angle was measured at least three times on each sample to ensure accuracy. Samples were tested for self-cleaning at a tilting angle of 8°. Figure 12a shows the self-cleaning test results of the superhydrophobic surface with water-based black ink. It can be seen that when the ink was dropped onto the surface from the top, the droplet rolled downwards to the bottom, leaving no trace on the surface, which indicated that the surface was stain-repellent. Another self-cleaning test was conducted on the superhydrophobic surface by staining with carbon black powder as shown in Figure 12b. The carbon black powder was randomly spread on the surface, and then water droplets were applied from the top of the sample. It is seen that the carbon black powder was easily removed, and no black residue was observed on the E-MTES/PFOTS surface. Therefore, the superhydrophobic E-MTES/PFOTS coating with self-cleaning capability would be suitable for practical applications to prevent contamination on copper surfaces. Figure 12c–f are schematic illustrations of the self-cleaning behavior. Figure 12c,d shows the movement of a water droplet on bare copper, where the droplet almost mixed with the contaminant due to the hydrophilic nature of the surface and the wettability of the substrate (CA < 90°). When the water droplet rolled on the superhydrophobic surface (Figure 12e,f), it rolled off together with the contaminant due to the water repellency of the superhydrophobic surface, leaving a clean surface after the experiment.

**Figure 13:**
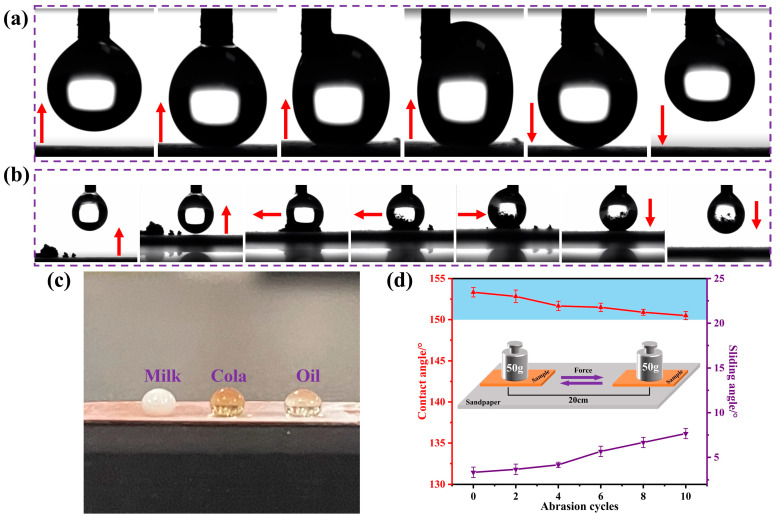
(**a**) The process of anti-sticking test of superhydrophobic surface (arrows indicating the platform moving direction). (**b**) The process of dust adsorption test by a water droplet on superhydrophobic surfaces (arrows indicating the platform moving direction). (**c**) Photographs of different liquid droplets on the coating surface (contact angles being 151°, 151.5°, and 129° respectively). (**d**) WCAs and sliding angle of the coating in relation to the abrasion cycle by sandpaper.

Figure 13a shows the process of the anti-sticking test of the superhydrophobic surface. The testing process was performed by squeezing the end of a fixed micro syringe allowing a water drop (size about 3 μm) to form at the tip and moving up and down the platform below to collect the drop. It was observed that even with forced contact of the water drop with the surface, the water drop was easily separated from the superhydrophobic surface without leaving any visible residues. During the contact, the water drop deformed and deflected to the side of the needle, indicating the non-sticking property of the surface. Figure 13b shows the testing process of dust removal by water droplets. The dust was absorbed by the water droplet and well removed from the surface, confirming the anti-sticking property of the surface. In addition to water, the coating was shown to exhibit excellent resistance to wetting by various liquids, including milk, cola, and vegetable oil (Figure 13c) with contact angles of 151°, 151.5°, and 129°, respectively. This indicates excellent self-cleaning properties of the coated surfaces, which would have potential applications for daily uses.

Wear and abrasion resistance pose a serious challenge for many practical applications of superhydrophobic surfaces under natural environmental conditions. The superhydrophobic coating was abraded using 1000-grit sandpaper (contact area 150 mm^2^) moving 20 cm distance per cycle under a load of 50 g. The abrasion resistance of the superhydrophobic E-MTES/PFOTS coating was evaluated on the basis of the measured wettability after 10 cycles of abrasion by sandpaper. As shown in Figure 13d, the WCA of the E-MTES/PFOTS coating surface did not change noticeably after 10 cycles and remained superhydrophobic (CA = 151°) with a sliding angle of about 8°. The good abrasion resistance of the 2-layer coating is due to the strong chemical bond between the basecoat and top-coat, as well as the complaint basecoat with a chemical bond on the copper substrate (as shown in Figure 7). The strength of these chemical bonds is much higher than any physical bonds, which demonstrates the intended design mechanism of the 2-layer coating system in this study.

### 3.7. Mechanisms of Chemical Bonding and Durability of Superhydrophobicity

**Figure 14:**
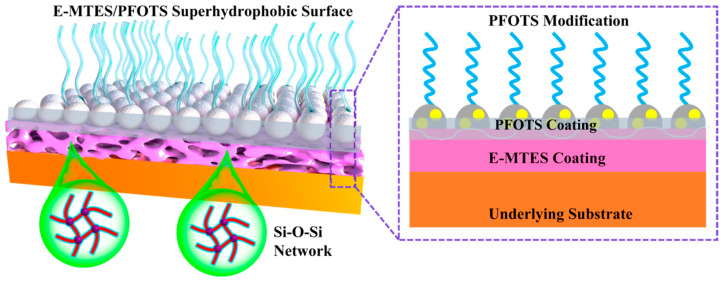
Illustration of the mechanism of the 2-layer coating structural design (**Left**), with enlarged interface structure (**right**).

The excellent abrasion resistance and durability of the superhydrophobicity of the developed 2-layer coating system are believed due to the strong chemical bonding and physical interlocking between the two layers, as well as the chemical bonding between the E-MTES layer and the substrate induced by the EAD process. Figure 14 illustrates the mechanism of the 2-layer coating structural design. As shown in the chemical reaction formula in Equations (1)–(3), the EAD process can accelerate the reduction of oxygen or increase the pH value near the electrode surface and induce more –OH groups by hydrolysis, which cause immediate condensation of the solute on the electrode surface and formation of the Si-O-Si network of the base-coat. Due to the concurrent deposition and reduction processes, a porous and rough surface is formed. This rough surface would provide local gaps for the penetration of the PFOTS top-coat. Meanwhile, the hydrolyzed silane groups in the PFOTS material would form a condensation reaction with the hydroxyl groups in the base coat. Therefore, strong bonding is established at the interfaces of the top coat and the base coat. The fluoro-side groups in the PFOTS chemical structure have high stability and tend to protrude on the surface providing a brush-like structure and long-lasting superhydrophobic properties. Some hydrophilic nano-silica particles were added into the PFOTES solution during hydrolysis, in which the nano-particle surfaces were grafted with the PFOTES’s fluoro-chains to further enhance the favorable surface morphology for superhydrophobicity.

### 3.8. Comparison with Other Research Work in Literature

Table 2 provides a comparison of the roughness, WCA, and reported properties of this work with other published results in literature. It is seen that most of the superhydrophobic coatings consist of silanes and fluoro-containing substances. Compared to the published results, this work has the advantage of using mild chemicals applied for copper substrate with a thicker and more durable coating.

## 4. Conclusions

In this work, a two-step process for obtaining superhydrophobic coatings was investigated for enhancing the protection of copper substrates. The base coat was prepared by the EAD process using MTES as the precursor of the sol-gel solution. It is shown that the surface topography, thickness, and surface roughness of the E-MTES films can be adjusted by controlling the deposition time and potential. The hierarchical surface topography of the EAD silica base coat ensured the penetration of the low surface energy PFOTS top-coat to be chemically bonded with the base coat and provided superhydrophobic properties. The E-MTES/PFOTS coatings exhibited excellent self-cleaning properties with WCAs of 153°, 151°, 151.5°, and 129° for milk, cola, and vegetable oil, respectively. The superhydrophobic E-MTES/PFOTS coating showed high chemical stability in corrosive solutions (acids and bases) and high abrasion resistance against sandpaper.

The developed 2-layer coating system demonstrated two distinctive benefits including: (1) the EAD process for a complaint uniform base-coat on the complex substrate with high adhesion strength; (2) a simple dip coating of PFOTS for the superhydrophobic top-coat with chemical bonding to the base-coat. The reported method would provide an environment-friendly and widely applicable approach for fabricating sol-gel silica-based superhydrophobic surfaces for enhancing the surface protection of metal substrates.

## Figures and Tables

**Table 1:** Copper Material chemical composition [wt%].

Element	Cu	Bi	Sb	As	Fe	Pb	S
Content	99.980	0.001	0.002	0.002	0.005	0.005	0.005

**Table 2:** Comparison of the properties and processes of several superhydrophobic coatings and this work by electrochemical techniques.

Substrate	Coating Material	Process Chemical Composition	Coating Methods	Contact Angle	Roughness	Thickness	Performance	Ref.
Al alloy	FP/SiO_2_	3.5 g SNP, 0.5 ml FAS-17, 1.2 mL KH560, 0.5 mL KH550 + FP	Two-step dipping	152.1°	-	-	Anti-corrosion, coating adhesion	[54]
Carbon steel	TPU/CNTs@SiO_2_	0.2 gc-CNTs, 50 mL EtOH/H_2_O + TEOS + F-17 + TPU	Simple coating	163°	-	-	Durability, chemical stability, self-cleaning and anti-fouling, anti-corrosion	[18]
Al alloy	TiO_2_-SiO_2_-silane	0.5 Macetylacetone and tetrabutyl titanate + 0.5 M tetraethyl orthosilicate + 0.2 M MTES, 50 mM FAS	Anodic anodization, Chemical deposition	165°	-	-	Stability, non-sticking, water repellency, self-cleaning, anti-icing	[55]
Ni/Cu	PFDS/ PNCA	15.6 g/L CuSO_4_·5 H_2_O, 17.6 g/L NiSO_4_·7 H_2_O, 14 g/L boracic acid, 3 g/L SDS + PDFS	electrochemical codeposition	163°	2.241 μm	-	Mechanical and chemical stabilities, self-cleaning corrosion assessment	[56]
Copper alloy	PFDS/ EDTA/ ACO	Copper alloy in 3.0 mol/L NaOH, 1 mol/L EDTA, 0.243 g Tris buffer + 0.16 g DA +5 g CuSO_4_ + 10 g H_2_O_2_, 1 wt% PFDS	Anodised	164.53°	-	-	Anti-scaling performance, corrosion resistance test	[57]
SS 306, ITO	E-SiO_2_	20 mL EtOH, 20 mL 0.1 M KNO_3_, 2 mL TEOS, 0.001 M HCl + DTMS	Two step electrochemical assisted deposition	>150°	>~1.0 μm	>~7.5 μm	-	[45]
MS	SiO_2_/ DTMS	20 mL 0.2 M KNO_3_, 80 mL EtOH, 2 mLTEOS, 2 mL DTMS	One step electrochemical assisted deposition	>150°	0–0.6 μm	1–5 μm	Corrosion assessment, iron dissolution determination	[46]
AZ31 Mg alloys	AZ31/e-DTMS	80 mL EtOH, 20 mL 0.2 M KNO_3_, 3 mL DTMS	One step electrodeposition	158°	-	15.6 μm	Self-cleaning performance, stability tests, corrosion behavior, hydrogen evolution test	[49]
MS	E-SiO_2_	50 mL EtOH, 5 mL TEOS, 50 mL 0.2 M NaNO_3_+ 3.0 vol.% DTMS	Two step method	155°	0–4 μm	0–20 μm	Corrosion test, abrasion resistance, stability	[43]
ITO	E-DTMS	2 mL DTMS, 80 mL EtOH, 20 mL 0.2 M KNO_3_ + 2 mL TEOS	One step sol–gel electrochemistry	>150°	0–2.7 μm	0–9 μm	Chemical stability, indentation tests	[39]
MS	SiO_2_	80/20 (*v*/*v*) EtOH/0.2 M KNO_3_+ 2.0 vol.% DTMS	One step electrodeposition	>150°	0–1.1 μm	1.3–5 μm	Stability, corrosion protection	[58]
Copper	E-MTES/ PFOTS	0.2 M KNO_3_, 200 mL EtOH, 50 mLMTES + 2.5 g PFOTS, 35 g propanol, 0.2 g SiO_2_	Two step electrochemical assisted deposition	153°	0.2–8.51 μm	0.7–78.26 μm	Stability tests, self-cleaning, durability, abrasion test, chemical stability, durability	This work

Abbreviations in Table: MS: Mild Steel, FP: Fluorocarbon paint; SNP: SiO_2_ nanoparticles; FAS-17: 1H,1H,2H,2H-perfluorodecyltriethoxysilane; KH560: γ-(2,3-epoxypropyloxy)-propyltrimethoxysilane; KH550: γ-aminopropyltriethoxysilane; CNTs: Carbon nanotubes; TPU: Thermoplastic polyurethane; TEOS: Tetraethyl orthosilicate; FAS: 1H,1H,2H,2H-perfluorodecyltrimethoxysilane; PFDS: 1H,1H,2H,2H-perfluorodecyltrimethoxysilane; PNCA: Prepared PFDS/NCA composite; SDS: Sodium dodecyl sulfate; EDTA: Ethylenediamine tetraacetic acid; ACO: Anodized copper oxide; DTMS: Dodeacyltrimethoxysilane.

## Data Availability

Not applicable.

## Supplementary Materials

The following supporting information can be downloaded at: https://www.mdpi.com/article/10.3390/ma16041417/s1, Figure S1: Cross-sectional morphology of E-MTES coatings thickness at different deposition times:(a) 100 s; (b) 200 s; (c) 400 s; (d) 600 s (prepared at −1.2V vs. Ag/AgCl); Figure S2: Cross-sectional morphology of E-MTES coatings thickness at different deposition potentials: (a) −1.0V; (b) −1.1V; (c) −1.2V; (d) −1.3V (deposition time: 600 s); Figure S3: SEM images and EDS elemental mapping results; Figure S4: EDS energy spectra and elemental compositions of E-MTES samples at different deposition times: (a) 100 s; (b) 200 s; (c) 400 s; (d) 600 s; Figure S5: FE-SEM images and EDS elemental mapping of the surface deposited at a typical deposition potential of −1.2V vs. Ag/AgCl for 600 s; Table S1: Film thickness values for different deposition durations; Table S2: Film thickness values for different deposition potential parameters.
