# Supplementary material for: Investigation of Electrochemical Assisted Deposition of Sol-Gel Silica Films for Long-Lasting Superhydrophobicity
Source: Materials (Basel). 2023 Feb 8;16(4):1417. doi: 10.3390/ma16041417 (PMC9968140; doi:10.3390/ma16041417)
Supplement: Supplementary file 1 [file materials-16-01417-s001.zip › materials-2106614-supplementary.pdf]

# Supplementary Information

# Investigation of Electrochemical Assisted Deposition of Sol-Gel Silica Films for Long-Lasting Superhydrophobicity

**Baoming Zhou, Yongling Wu * and Hongyu Zheng ***

Centre for Advanced Laser Manufacturing (CALM), School of Mechanical Engineering, Shandong University of Technology, Zibo 255000, China

* Correspondence: ylwu06@sdut.edu.cn (Y.W.); zhenghongyu@sdut.edu.cn (H.Z.)

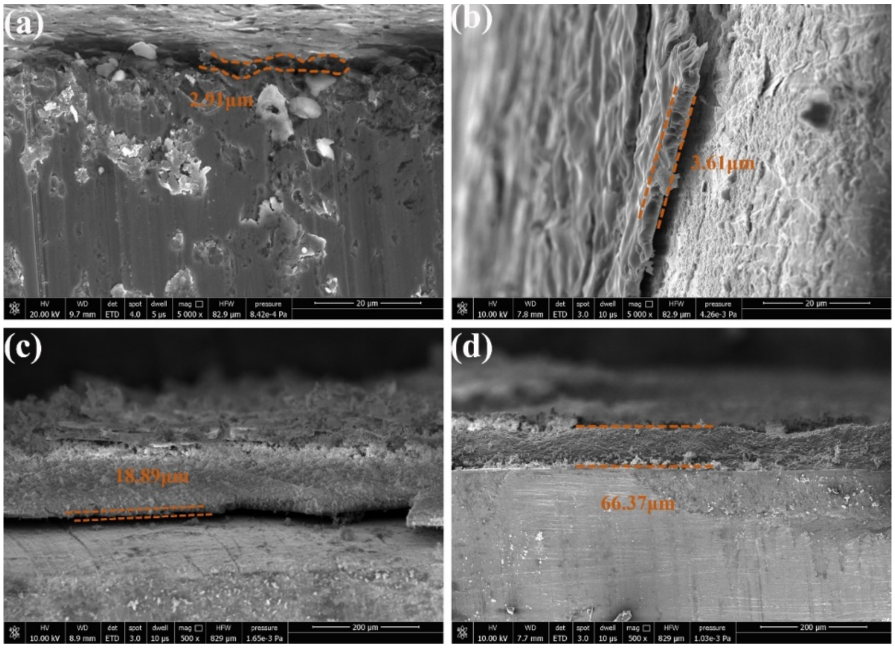


**Figure S1.** Cross-sectional morphology of E-MTES coatings thickness at different deposition times:(a)100s; (b)200s; (c)400s; (d)600s (prepared at -1.2V vs. Ag/AgCl).

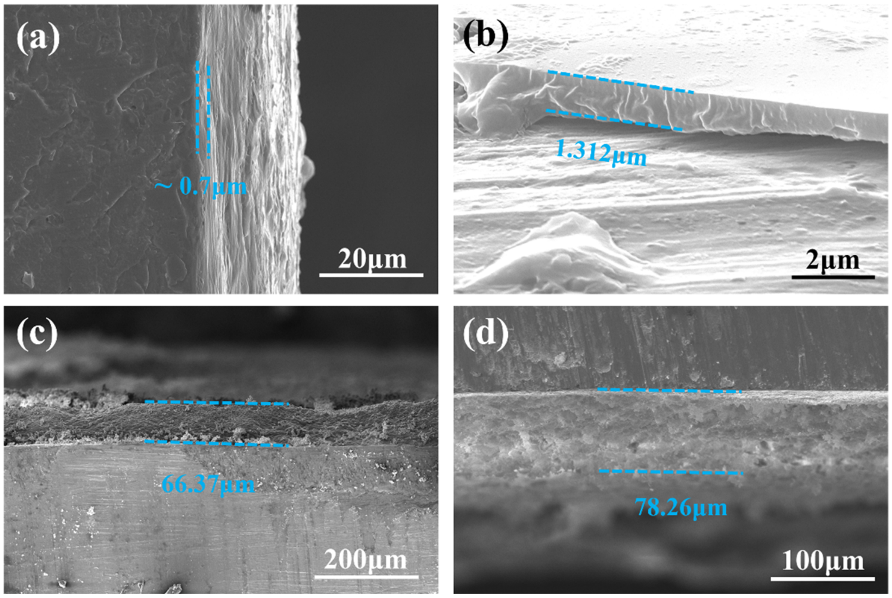


**Figure S2.** Cross-sectional morphology of E-MTES coatings thickness at different deposition potentials: (a)-1.0V; (b)-1.1V; (c)-1.2V; (d)-1.3V (deposition time: 600s).

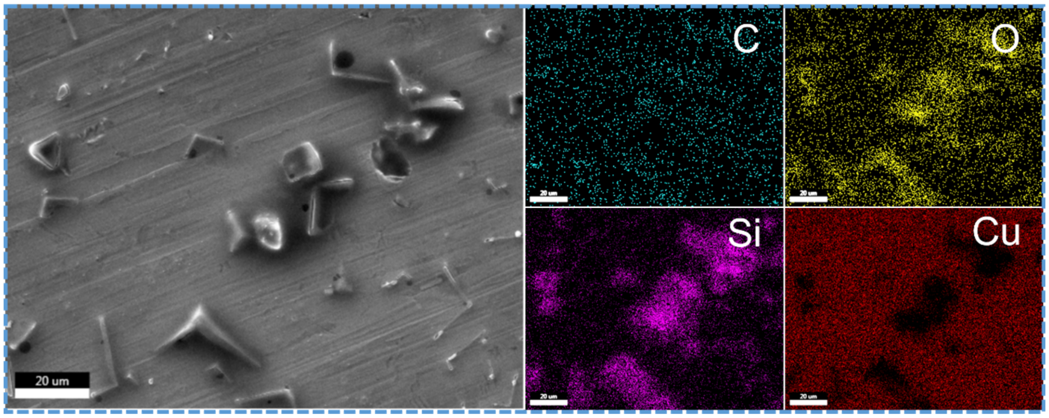


**Figure S3.** SEM images and EDS elemental mapping results.

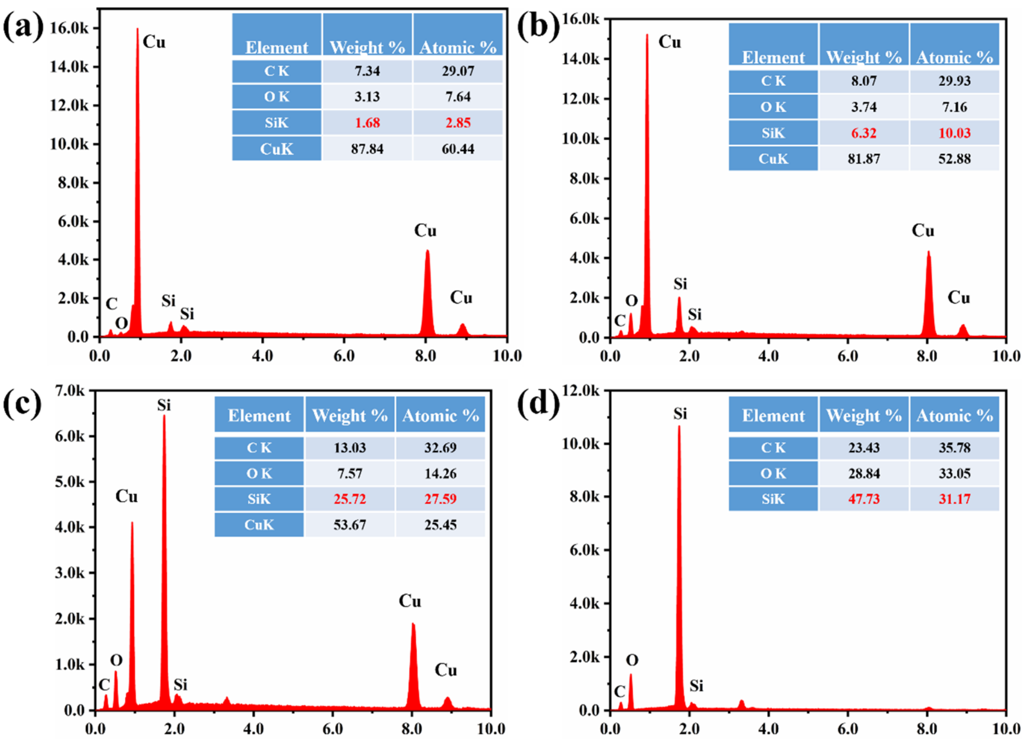


**Figure S4.** EDS energy spectra and elemental compositions of E-MTES samples at different deposition times: (a) 100s; (b) 200s; (c) 400s; (d) 600s.

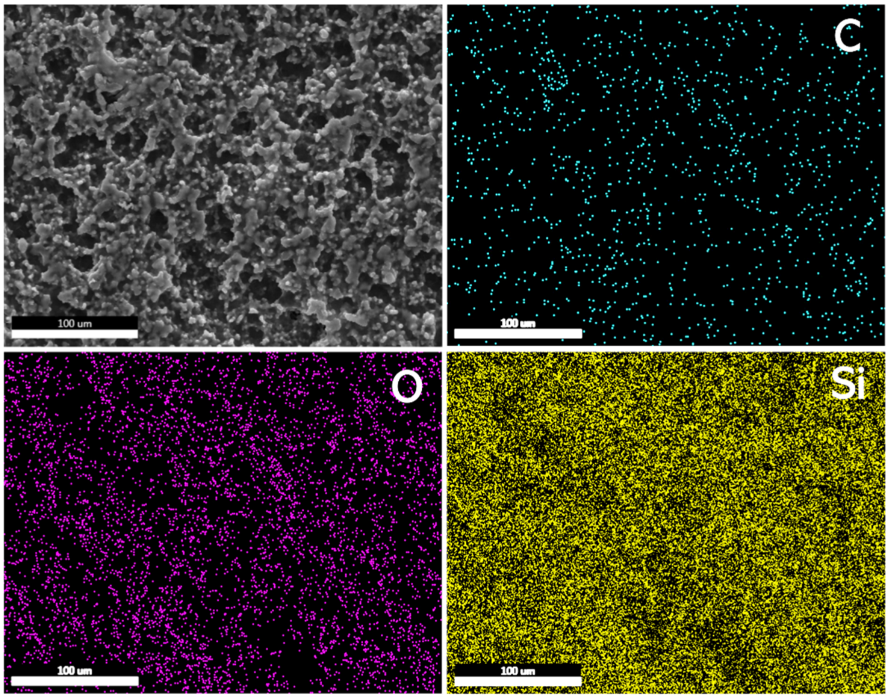


**Figure S5.** FE-SEM images and EDS elemental mapping of the surface deposited at a typical deposition potential of -1.2V vs. Ag/AgCl for 600s.

**Table S1.** Film thickness values for different deposition durations.

| Parameter | All processes at -1.2V vs. Ag/AgCl | | | |
|---|---|---|---|---|
| Deposition time | 100s | 200s | 400s | 600s |
| Thickness/μm | 2.91 | 3.61 | 18.89 | 66.37 |

**Table S2.** Film thickness values for different deposition potential parameters.

| Parameter | The deposition time is 600s for all samples | | | |
|---|---|---|---|---|
| Deposition potential | -1.0V | -1.1V | -1.2V | -1.3V |
| Thickness/μm | 0.7 | 1.312 | 66.37 | 78.26 |
